# Main-chain mutagenesis reveals intrahelical coupling in an ion channel voltage-sensor

**DOI:** 10.1038/s41467-018-07477-3

**Published:** 2018-11-29

**Authors:** Daniel T. Infield, Kimberly Matulef, Jason D. Galpin, Kin Lam, Emad Tajkhorshid, Christopher A. Ahern, Francis I. Valiyaveetil

**Affiliations:** 1 Department of Molecular Physiology and Biophysics, Iowa Neuroscience Institute, University of Iowa, Iowa City, IA 52242 USA; 2 Program in Chemical Biology, Department of Physiology and Pharmacology, Oregon Health Sciences University, Portland, 97239 OR USA; 3 Department of Physics, University of Illinois at Urbana-Champaign, Urbana, IL 61801 USA; 4 NIH Center for Macromolecular Modeling and Bioinformatics, Beckman Institute for Advanced Science and Technology, University of Illinois at Urbana-Champaign, Urbana, IL 61801 USA; 5 Department of Biochemistry, Center for Biophysics and Quantitative Biology, University of Illinois at Urbana-Champaign, Urbana, IL 61801 USA

## Abstract

Membrane proteins are universal signal decoders. The helical transmembrane segments of these proteins play central roles in sensory transduction, yet the mechanistic contributions of secondary structure remain unresolved. To investigate the role of main-chain hydrogen bonding on transmembrane function, we encoded amide-to-ester substitutions at sites throughout the S4 voltage-sensing segment of *Shaker* potassium channels, a region that undergoes rapid, voltage-driven movement during channel gating. Functional measurements of ester-harboring channels highlight a transitional region between α-helical and 3_10_ segments where hydrogen bond removal is particularly disruptive to voltage-gating. Simulations of an active voltage sensor reveal that this region features a dynamic hydrogen bonding pattern and that its helical structure is reliant upon amide support. Overall, the data highlight the specialized role of main-chain chemistry in the mechanism of voltage-sensing; other catalytic transmembrane segments may enlist similar strategies in signal transduction mechanisms.

## Abstract (web summary)

The helical transmembrane segments of membrane proteins play central roles in sensory transduction but the mechanistic basis for their function remains unresolved. Here the authors identify regions in the S4 voltage-sensing segment of *Shaker* potassium channels where local helical structure is reliant upon backbone amide support.

## Introduction

Membrane proteins catalyze the passage of biological information. Central to this function is the ability to transduce a physiological stimulus, such as a change in membrane voltage, tension, temperature, or ligand binding, into a protein conformational change. Transmembrane segments of eukaryotic membrane proteins adopt primarily helical secondary structure^1^. These helices tend to be much longer than those in soluble proteins and often contain kinks, breaks, or segments of alternative helical content, such as a 3_10_- or a π-helix^2–5^. These features display distinct patterns of hydrogen bonds, the strength of which is dependent on the local environment^6^. Disruptions in the transmembrane helical segments have been proposed to act as hinges or as switches during the conformational changes that are essential for the function of these proteins^7–11^. Despite their potential roles in membrane protein function, data revealing the energetic contributions of main-chain hydrogen bonding towards a functional output are limited^9^.

**Fig. 1:**
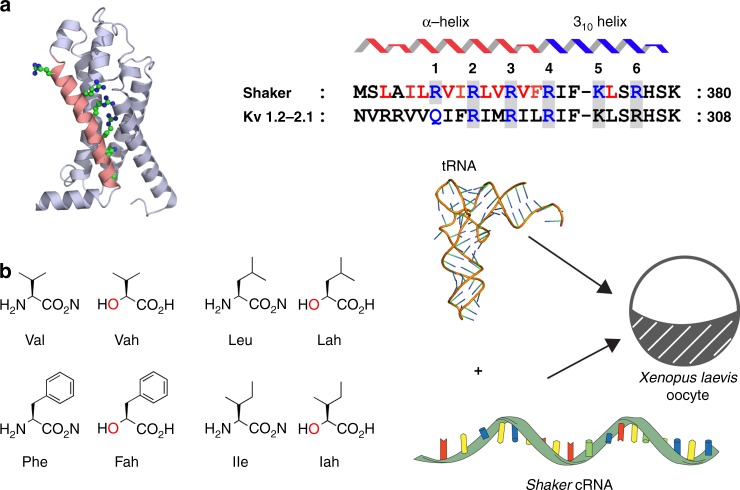
Main chain mutagenesis in a voltage-gated potassium channel voltage-sensor. **a** Structure of the voltage-sensing domain (VSD) of the Kv1.2/2.1 chimera (pdb 2R9R). The S4-helix is in pink with side-chains for conserved Arg and Lys residues shown as sticks. Right, sequence alignments of the S4 sequence. Residues mutated in Shaker are shown in red. The α-helical (red) and 3_10_-helical (blue) regions in the Kv1.2/2.1 structure are shown above the alignment. **b** Structures of amino acids (aa) and α-hydroxy acids (ah): Vah hydroxyl-isovaleric acid, Lah 2-hydroxy-4-methylpentanoic acid, Fah 3-phenyl lactic acid, Iah 2-hydroxy-3-methylpentanoic acid. Right, these are ligated to the tRNAs (pdb 2ZNI) for nonsense suppression. *Shaker* cRNA containing a UAG stop codon at the suppression site is co-injected with an orthogonal amber (TAG) suppressor tRNA chemically ligated to an amino acid or α-hydroxy acid (red)

The intensely studied process of voltage-dependent ion channel gating provides a unique opportunity to quantify the structural and functional effects of hydrogen bond manipulation^12,13^. Voltage-dependent ion channels are ancient membrane proteins that interconvert transmembrane potential into ionic flux, thus shaping the action potentials that form the basis for electrical communication^14^. These proteins are comprised of four homologous domains, each with six helical transmembrane segments, termed S1–S6. A modular voltage-sensing domain (VSD) is formed by S1–S4, and the central pore and gates are established by S5–S6 and a re-entrant pore loop^15^. The S4 segment in the VSD contains highly conserved basic residues positioned at every third site along the length of the helix^16^ (Fig. 1a). These so-called gating charges mobilize the S4 segment in an outward trajectory of 10–15 Å in response to membrane depolarization, leading to conformational changes in the pore domain that regulate the ionic flow across the membrane^17–22^. This movement includes passage of cationic residues on S4 from one aqueous environment (intracellular lumen) to another (extracellular space) through a narrow hydrophobic gasket^17^. Another feature of this segment is an unusually long 3_10_-helical component found in voltage-gated channels and other members of this super family (Fig. 1a, right)^23–29^. The regions of the channel that are α-helical versus 3_10_-helical differ in these structures, and the mechanistic significance of these differences is not known. The high-resolution crystal structure of the Kv1.2/2.1 chimera, most closely related to the *Shaker* K^+^ channel used in this study, shows that in the activated state, the N-terminal half of the Kv S4 is an α-helix, while the C-terminal portion is a 3_10_-helix, which are termed the S4a and S4b helical segments, respectively^23,30,31^. The S4 segment provides an ideal venue to probe the energetic contributions of structural perturbations as the effects are readily quantified using well-developed electrophysiological assays.

Here, we use in vivo nonsense suppression to determine the energetic contributions of main-chain structure in the S4 segment to voltage-dependent gating of *Shaker* potassium channels. Specifically, we encode α-hydroxy acids at several individual positions along the transmembrane segment. This results in the substitution of single backbone amides with esters and the corresponding loss of a specific hydrogen bond in the helix. The biochemical and electrophysiological data show that these derivatives are tolerated in the *Shaker* VSD and they support the robust expression of engineered voltage-gated potassium channels with a range of gating phenotypes. We find that the functional effect of the ester substitution is highly position dependent. The greatest effect of hydrogen bond ablation is at a residue between R3 and R4, at the transition between the α-helical and 3_10_-helical segments of the S4 in the activated conformation of the voltage-sensor. We use molecular dynamics (MD) simulations to gain a more detailed view of structural perturbations caused by the introduction of the main-chain ester in this segment. These data establish functional nonequivalence among hydrogen bonds within the voltage-sensor, and highlight an S4 region where hydrogen bonding is especially critical for voltage-dependent gating.

## Results

### Incorporation of amide-to-ester substitutions

To probe the role of the main-chain hydrogen bonding in voltage gating, we used amide-to-ester substitutions along the S4 segment (Fig. 1b). Ester linkages have bond lengths and bond angles that are similar to amide bonds, they prefer the trans geometry, and they have a high energy barrier for rotation around the bond (10–15 kcal/mol for an ester bond compared to 18–21 kcal/mol for an amide bond)^32,33^. However, the ester oxygen, unlike the amide N-H, cannot act as a hydrogen bond donor, and therefore the ester substitution results in the deletion of a protein main-chain hydrogen bond. A key advantage of this atomic manipulation is that it does not alter the amino acid side-chain.

**Fig. 2:**
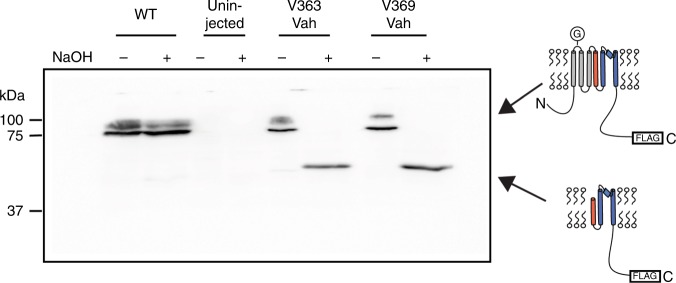
Hydrolysis of α-hydroxy encoded channels. Western blot of crude membrane preps of oocytes injected as labeled. WT channels cannot be hydrolyzed by base (NaOH, see Methods), but V363Vah and V369Vah are cleaved at the ester bond with base addition. Full-length channels contain a conserved glycosylation site (G), producing a two-band pattern. Channels are detected with a C-terminal FLAG tag

Ester substitutions were introduced via in vivo nonsense suppression of amber (TAG) stop codons (Fig. 1b, right)^34^. Briefly, complementary RNA (cRNA) for the *Shaker* channel containing the introduced amber (TAG) mutation is co-injected into a *Xenopus* oocyte along with an amber suppressor transfer RNA (tRNA). These tRNAs have been acylated with an α-hydroxy acid (Vah, Lah, Fah, or Iah, Fig. 1b, left), resulting in the encoding of an ester linkage at the TAG site. To confirm the introduction of the α-hydroxy acids at representative sites, we made use of the fact that proteins harboring encoded α-hydroxy acids are susceptible to alkaline hydrolysis, while proteins with natural amide main-chains are not^35^. For these experiments, we incorporated a FLAG tag at the C-terminus to facilitate detection of the *Shaker* protein by Western blotting. The FLAG tag did not affect channel function (Supplementary Figure 1). Figure 2 shows that wild-type (WT) *Shaker* channels are resistant to NaOH treatment, whereas channels with Vah show complete hydrolysis at both sites, indicating stringent encoding of the intended unnatural α-hydroxy acid under the expression conditions used for subsequent functional analysis.

### Functional effects of ester substitutions in S4

**Fig. 3:**
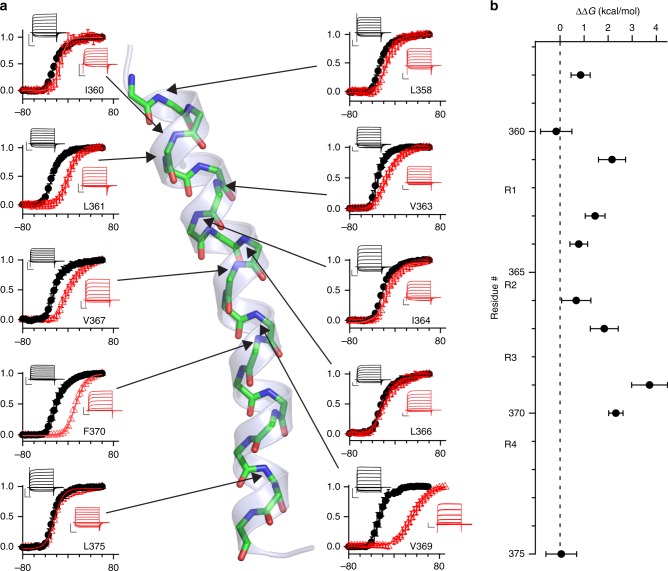
Functional nonequivalence of ester substitutions in the S4 segment. **a** Conductance–voltage (*G*–*V*) relationships for amino (WT, black) and hydroxyl acids (red) encoded at the indicated sites in S4. Solid lines represent Boltzmann fits to the data obtained with 5 mV steps. Insets show representative potassium current traces elicited with depolarizations from −80mV to +60 mV in 10 mV steps. Full-size currents and pdCpA-negative control traces can be found in Supplementary Figure 2. Scale bars equal 5 μA and 50 ms for current size and duration, respectively. **b** Free energy change on channel gating by site. All data shown in **a**, **b** are mean ± standard deviation from 3 to 13 oocytes. Data were repeated from at least two separate batches of cRNA and suppressor tRNA

We next incorporated ester substitutions at several positions in the S4, from L358 at the extracellular end to L375 at the intracellular end, and interrogated channel function using the two-electrode voltage clamp (TEVC) technique to determine the voltage-dependent gating for each mutant (Fig. 3a, red). Site-specific impacts on channel function provide a direct energetic metric on the functional consequence of H-bond removal in this transmembrane segment. At each position, control suppression experiments are shown with the WT amino acid (Val, Ile, Leu, or Phe) appended to the suppressor tRNA (Fig. 3a, black). Additionally, the potential for spurious bleed-through of the introduced stop codon was tested by co-injection of cRNA with an equivalent concentration of unacylated, full-length tRNA (pdCpA or pCA) control) (Supplementary Figure 2). Under the indicated experimental conditions, the suppression via tRNA acylated with both the amino- or α-hydroxy acid variants produced robust voltage-dependent outwardly rectifying potassium currents (Fig. 3a, inset), whereas unacylated tRNA (pCA control) did not (Supplementary Figure 2). As expected, suppression with the natural amino acid produces with conductance–voltage (*G*–*V*) values similar to the native channel (Table 1). In contrast, the ester mutants show a range of equilibrium values from normal to strongly depolarized, indicating position dependent effects of the amide-to-ester substitution on voltage gating. Ester substitution at position V369, between R3 and R4 at the α-helix/3_10_-helix junction, results in a maximal effect on voltage gating; the *G*–*V* curve is shallowed and shifted by nearly 60 mV to the right. The thermodynamic effects of all 10 ester mutants are formalized as free energy (∆∆*G*) effects on gating of main-chain ester substitution by site (Fig. 3b)^36^. Here, the positional dependence is apparent, with the most significant perturbation within the S4 segment centered at V369. For five positions, we performed additional experiments in order to analyze channel deactivation kinetics, which revealed two main insights. First, at V363 and L366, where ester substitutions minimally perturbed the *G*–*V* curves, deactivation kinetics were normal (Supplementary Figure 3), suggesting the absence of compensatory kinetic changes at these positions. Second, ester mutations at positions that had progressively larger effects on the *G*–*V* curves (V367, F370, and V369) tended to also speed up deactivation (Supplementary Figures 3 and 4). While the deactivation rate of the V369 ester variant was significantly accelerated compared to WT channels, the activation rate was not significantly affected (Supplementary Figure 4). These data suggest that the loss of the main-chain hydrogen bond at V369 destabilizes the structure of the open state of *Shaker*.

**Table 1:** Gating parameters with extracellular K^+^

	*V*_d_	*z*		*V*_d_	*Z*
WT	−25.9 ± 3.4 (10)	3.4 ± 0.7 (10)			
L358Leu	−25.6 ± 2.3 (7)	3.2 ± 0.3 (7)	L358Lah	−14.4 ± 3.6 (7)	3.1 ± 0.6 (7)
I360Ile	−22.2 ± 2.6 (7)	2.7 ± 0.5 (7)	I360Iah	−13.9 ± 4.5 (5)	4.8 ± 0.9 (5)
L361Leu	−28.1 ± 3.0 (8)	3.2 ± 0.7 (8)	L361Lah	1.3 ± 3.7 (13)	3.0 ± 0.4 (13)
V363Val	−28.6 ± 3.8 (4)	3.3 ± 0.2 (4)	V363Vah	−14.7 ± 5.1 (5)	2.2 ± 0.3 (5)
I364Ile	−21.3 ± 0.6 (3)	2.9 ± 0.3 (3)	I364Iah	−11.2 ± 5.2 (7)	2.5 ± 0.6 (7)
L366Leu	−23.5 ± 2.5 (8)	2.8 ± 1.0 (8)	L366Lah	−17.8 ± 1.9 (11)	2.0 ± 0.5 (11)
V367Val	−24.0 ± 4.1 (7)	3.1 ± 0.7 (7)	V367Vah	1.4 ± 5.3 (8)	2.3 ± 0.5 (8)
V369Val^a^	−25.5 ± 6.0 (9)	3.8 ± 0.6 (9)	V369Vah^a^	33.0 ± 7.9 (10)	1.9 ± 0.2 (10)
F370Phe	−21.5 ± 2.9 (5)	2.8 ± 0.3 (5)	F370Fah	13.6 ± 2.0 (7)	2.9 ± 0.2 (7)
L375Leu	−26.0 ± 2.6 (8)	3.5 ± 0.6 (8)	L375Lah	−23.4 ± 3.8 (14)	3.8 ± 0.6 (14)

Mean values are shown ± standard deviation with *n* values within parentheses. Tail currents at −50mV were used to determine parameters except for V369 mutations

^a^Ionic current normalized to driving force were used to determine *G*–*V*s for V369Val and V369Vah

### Assessing impact of H-bond ablation on S4 translocation

The displayed *G*–*V* relationships report on channel opening, a process that is mechanistically downstream from voltage-sensor activation. In order to ascertain direct information of the effect of ester substitution on S4 function, we measured the transient gating currents directly generated by the movement of S4 charges through the transmembrane electric field. In order to measure the gating currents, the W434F mutation was made that essentially eliminates potassium conductance^37^. We focused this examination on S4 positions V363 and V369 because these mutations had divergent effects on voltage gating: ester substitution at V363 caused mild effects, while ester substitution at 369 resulted in the largest energetic effect (Fig. 3).

**Fig. 4:**
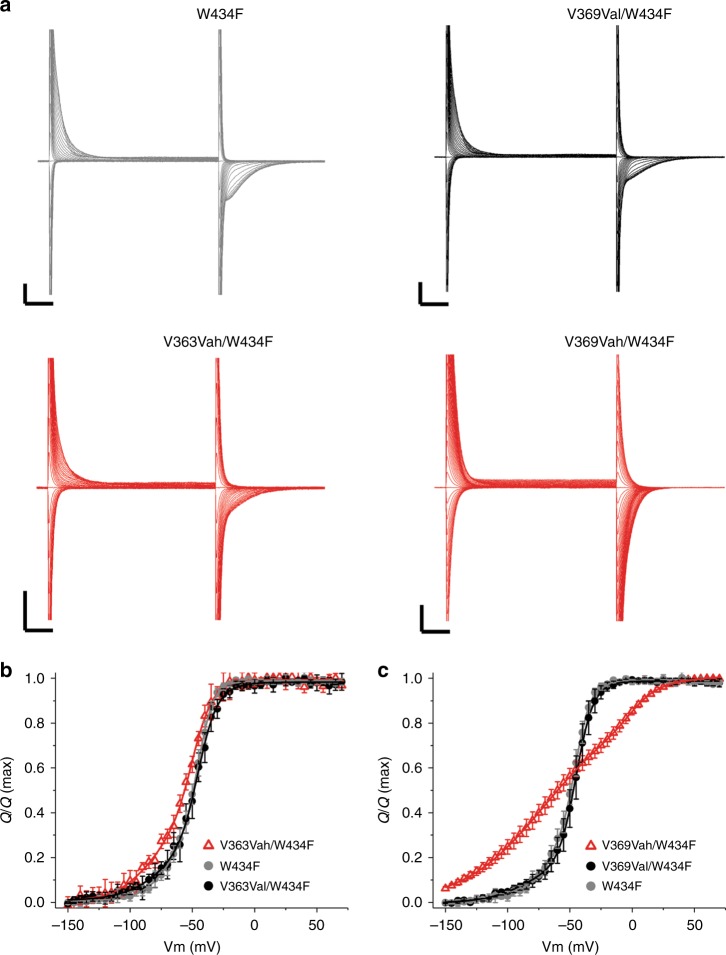
A backbone ester mutation between R3 and R4 alters the movement of S4. **a** Exemplar traces showing gating currents and capacitance in response to membrane depolarizations for *Shaker* W434F and W434F/V369TAG co-injected with valine acylated tRNA (top) as well as W434F/V363TAG and W434F/V369TAG co-injected with valine α-hydroxy tRNA (bottom). For each trace, voltages between −150 and 0 mV are displayed, with the exception of V369Vah/W434F, wherein −150 to +50 mV is shown. Scale bars denote 3 µA (*Y-*axis) and 5 ms (*X*-axis). **b** *Q*-*V* relationship for V363Vah/W434F (red) compared to either the W434F or the V363Val/W434F control. Gating *Q* was extracted from traces by subtracting the linear capacitance from the integrated signal from the OFF currents^39^, Supplementary Figure 5. **c** Same as in **b**, but comparing *Q*-*V* relationships between V369Vah/W434F and either W434F or the V369Val/W434F control. All data shown in **b,** **c** are mean ± standard deviation from four to eight oocytes. Note the shallowing of the curve generated by the ester mutation at position 369, as well as the two visible components of gating charge

Representative capacitive current recordings are shown in Fig. 4a for each indicated channel type. The nonlinear gating current (*Q*) was isolated via offline subtraction of the entire linear oocyte capacitance from the integrated OFF transient current, to reveal gating charge–voltage (*Q*–*V*) relationships (Supplementary Figure 5)^38^. The method generated *Q*–*V* curves that were in very close agreement with those reported in previous studies (Fig. 4b, c, gray lines)^38,39^. Injection of unacylated tRNA with W434F-V363TAG or W434F-V369TAG cRNA yielded negligible nonlinear charge displacement (Supplementary Figure 6). Rescue of both W434F-V363TAG and W434F-V369TAG with Val-tRNA closely recapitulated the *Shaker* W434F gating currents (Fig. 4b, c, black lines). Vah substitution at V363 produced modest changes in the *Q*–*V* distribution (Fig. 4a, b, left). By contrast, substitution of Vah at 369 produced a drastically altered *Q*–*V* relationship (Fig. 4c). Specifically, compared to WT channels, V369Vah channels had a significant amount of charge move at hyperpolarized potentials (<−100 mV). In addition, the phases of charge movement associated with *Shaker* activation were separated^40^, with the depolarized component being significantly right-shifted compared that of W434F controls (Fig. 4c, Supplementary Table 1). Thus, although removal of a hydrogen bond at V369 enabled a significant component of S4 movement at negative potentials (Fig. 4c), this movement was insufficient for channel activation, which occurred in the voltage range of the second, more depolarized, component (Table 1). Finally, W434F-V369Vah *Shaker* featured a weakened overall voltage dependence of S4 movement in response to depolarization, as evidenced by decreased slopes for both components of charge movement (Supplementary Table 1).

### Molecular dynamics highlights an S4 transition region

**Fig. 5:**
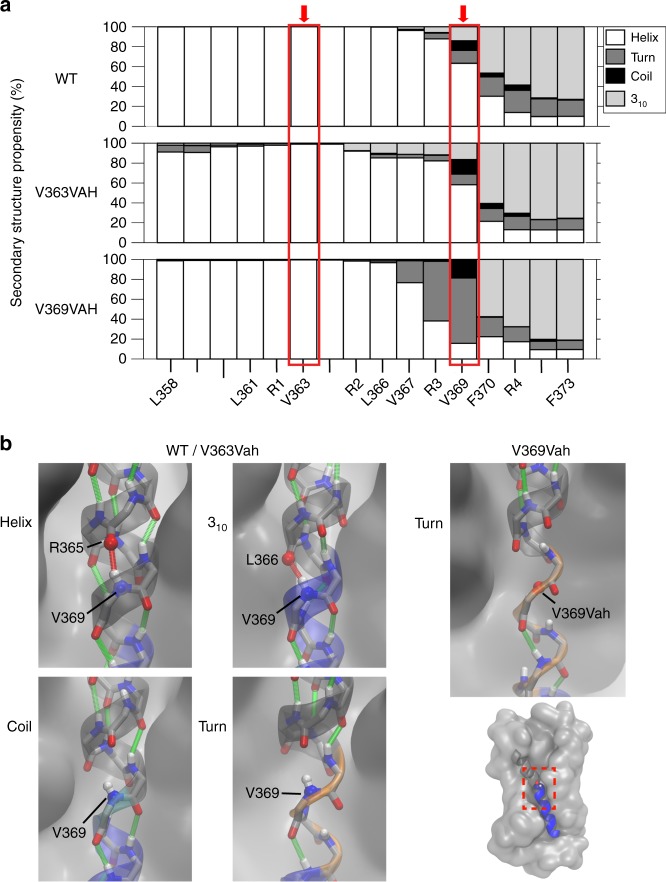
Simulations identify secondary structure sampling at the S4 transition region. **a** Helical content of the S4 segment for WT, V363Vah, and V369Vah shown on top, middle, and bottom panels, respectively. Main-chain structure at V363 and V369 highlighted by red boxes. Top, WT S4 maintains the α-helical (white) and 3_10_ elements at the N- and C-termini, and highlights helical sampling at V369—the transition point. Middle, V363Vah recapitulates WT S4 structural elements. Bottom, V369Vah displays a localized loss of helical structure. **b** Representative snapshot images from the simulations for WT, 363Vah (left), and 369Vah. Main-chain structure is represented by the transparent cartoon overlay and main-chain H-bonds are indicated by red and green lines

As the structure of the *Shaker* K^+^ channel is presently not available, the active state structure of the K_v_1.2/2.1 chimera channel was used for MD simulations^23^. We carried out four independent 150ns-long equilibrium simulations on the activated VSD for the WT and for channels with an ester substitution at I291 or I297 (positions corresponding to V363 and V369 in *Shaker*). The channels were simulated in a 1-palmitoyl-2-oleoyl-*sn*-glycero-3-phosphocholine (POPC) lipid bilayer. In the simulation of the WT channel, the upper half of S4 (*Shaker* residues 358–366) maintained an α-helical secondary structure, while the lower half adopted a 3_10_ helical conformation 60–80% of the time (Fig. 5a). The α- to 3_10_-helical transition at R368 and V369, as observed in the crystal structure, was preserved during the equilibrium simulations. During the simulations, the transition region sampled hydrogen bonding patterns corresponding to α- and 3_10_-helical conformations. Further, the transition region also sampled turn and coil conformations in which the H-bonding was disrupted. Representative images of H-bonding patterns are shown in Fig. 5b and in Supplementary Movies 1–3.

The simulation of the V363Vah channel showed that the H-bond removal at V363 due to the ester substitution did not perturb helical structure (Fig. 5a, middle). The minimal structural effect of the 363 ester substitution observed in the MD simulations is consistent with the modest functional perturbation observed with the V363 ester substitution. In contrast, simulations of the V369Vah channel showed a loss of helical content in the α-helix/3_10_-helix transition region that was tightly localized: the α-helical nature of the upper S4a segment and the 3_10_-helical nature of the lower S4b segment were maintained (Fig. 5b, right, and Supplementary Movie 2). In addition, no significant interactions between the introduced esters in S4 and nearby domains were observed in the simulations, suggesting the functional effects of ester substitution are primarily due to local changes in the structure of the S4 (Supplementary Figure 7).

## Discussion

Here we report the application of main-chain mutagenesis to the S4 segment in *Shaker* potassium channels. The conformational changes undergone by this helix control the gating of voltage-gated K^+^, Na^+^, Ca^2+^, and H^+^ channels, as well as the enzymatic activity of voltage-dependent phosphatases^13,41^. We find that main-chain ester substitutions are broadly tolerated in this region and produce potassium channels which display a range of functional phenotypes. Natural amino acids vary greatly in terms of helical propensity; there are myriad examples where conventional (side-chain) mutagenesis has been utilized to manipulate secondary structure. By contrast, the approach deployed here allows for the direct assessment of the energetic contributions within a transmembrane helical segment, in the absence of changes in side-chain chemistry. This approach is especially well suited for study of the tight molecular environment in the VSD, a region known to be highly sensitive to side-chain mutagenesis.

Interestingly, there was no obvious correlation with microenvironment polarity and the magnitude of effects of the ester substitutions on gating; all sites tested, except for L358 and L375, are proposed to traverse the entire electric field and inhabit a variety of dielectric environments as they transit the hydrophobic gating charge transfer center between aqueous vestibules in the VSD. While there were functional affects at several sites along the S4, the most perturbed voltage-dependent activation in response to ester substitution was at 369, between R3 and R4 and our data show that removal of the H-bond accelerates deactivation. (Fig. 3, Supplementary Figure 4). What is the structural mechanism for this effect? The S4 helix of *Shaker* and of other voltage sensors likely moves in two discrete steps^40,42^. Fitting of the gating current data for V369Vah (Fig. 4) reveals that the gating charge associated with the first step (Q1) has a similar midpoint voltage to WT (Supplementary Table 1). However, the slope of Q1 is shallow relative to WT and the fraction of total charge that moves at this step is increased (~30% versus ~80% for WT and V369Vah, respectively). These effects are consistent with the possibility that elimination of the H-bond associated with the V369 amide allows for the emergence of S4 conformations, which are rare or absent in this voltage range in the native protein. In turn, the data also suggest that the steep voltage dependence of charge movement in the WT protein is dependent on secondary structure in this region. In contrast to Q1, the midpoint voltage for movement of Q2 of V369Vah is shifted >40 mV in the depolarized direction (Supplementary Table 1), where it is well correlated with channel opening in this mutant. Removal of the hydrogen bond at 369 therefore results in perturbed function of the latter gating charges (presumably R3 and R4), which control channel activation^43,44^. Overall, the gating current data suggest the mechanism of the enhanced deactivation kinetics of V369Vah is due to an energetically unfavorable, and therefore an unstable, active S4 conformation.

This mechanism is supported and illustrated by the MD simulations performed in this study. We find that in the fully activated WT VSD, the secondary structure around position 369 is dynamic (Fig. 5a top, Supplementary Movie 1). This is pursuant to this region serving as a transition point in the active state between S4a (α-helical) and S4b (3_10_). Simulation of the ester mutation at this position reveals that main-chain H-bonding in this transition region may be essential for maintenance of secondary structure, whereas in other positions (e.g., V363) it may not be as crucial (Fig. 5a, bottom).

It has been proposed that as S4 moves during voltage gating there is a shift in the secondary structure from α- to 3_10_- helical conformation in the stretch of S4 that is buried in the gating charge transfer center^15,23,30,45–47^. The approach used here does not explicitly test the possibility of conversion between 3_10_ versus α-helical states, per se. However, the observation that some ester substitutions within the S4 fail to elicit major effects on voltage-dependent gating is consistent with the possibility that during gating, such an α-to-3_10_ transition is driven by the microenvironment as opposed to main-chain hydrogen bonding. If in fact such a conversion occurs within the apolar gating charge transfer center, it might be predicted to be a low energy conformational change^48^.

The functional and MD data presented herein also suggest that the stability of the active conformation of the WT VSD is highly reliant on the individual backbone H-bonds within the vicinity of R3–R4. In this work, the use of amide-to-ester mutagenesis allowed us to probe the role of main-chain hydrogen bonds of the S4 segment, and we anticipate that these approaches will be widely beneficial for the examination of helical energetics within other transmembrane proteins.

## Methods

### Molecular biology and in vivo nonsense suppression

*Shaker* Δ6-46 in the pAMV vector^49^, referred to as WT throughout this paper, was kindly provided by Dr. Ming Zhou (Baylor College of Medicine). Point mutations in the *Shaker* channel gene were generated using site-directed mutagenesis or Gibson Assembly^50^ and confirmed by sequencing (Supplementary Table 3). cRNA was transcribed using the mMessage mMachine Kit (Thermo Fisher) and purified using the RNeasy Kit (Qiagen). *Xenopus laevis* oocytes were kindly provided by Dr. Michael Danilchik (OHSU, protocol # IP00000214), Dr. Pablo Artigas (Texas Tech University, protocol # 11024), or purchased from Ecocyte Bioscience. For incorporation of amide-to-ester substitutions, the oocytes were typically injected with 50 nl of a mixture containing ~0.5–25 ng of *Shaker* cRNA with the amber stop codon and ~5–125 ng of an amber suppressor tRNA acylated with the respective α-hydroxy acid (Lah, Vah, Fah, or Iah). Vah = hydroxyl-isovaleric acid, Lah = 2-hydroxy-4-methylpentanoic acid, Fah = 3-phenyl lactic acid, Iah = 2-hydroxy-3-methylpentanoic acid. In all cases, controls were done where the *Shaker* cRNA was co-injected with the suppressor tRNA acylated with the corresponding amino acid (Ile, Leu, Val, or Phe and referred to as the native control) or with the suppressor tRNA by itself, lacking an amino or hydroxy acid (referred to as the pCA control).

### Synthesis of suppressor tRNA

The synthesis of isoleucine/phenylalanine/leucine/valine α-hydroxy-phosphodesoxycytosine phosphoadenosine (Iah/Fah/Lah/Vah-pCA) was achieved as follows. All solvents and reagents were supplied by Sigma-Aldrich and were used as is unless explicitly stated. pCA was obtained through GE Healthcare/Dharmacon. Dry nitrogen was supplied by Praxair and passed through two moisture scrubbing columns of dry calcium sulfate (Drierite) prior to use. High-performance liquid chromatography (HPLC) analyses were performed on a Waters 1525 Binary HPLC pump equipped with a Waters 2998 Photodiode Array Detector, employing Sunfire C18 analytical (3.5 µm, 4.6 mm × 150 mm, 0.8 ml/min) or preparative (5.0 µm, 19 mm × 150 mm, 10 ml/min) columns and Empower software, buffers were drawn in linear gradients from 100% A (50 mM ammonium acetate) to 100% B (acetonitrile) over 30 min. Ultraviolet–visible (UV–vis) spectra for concentration determinations were recorded on a Thermo Scientific Nanodrop 2000C spectrophotometer. Mass spectra were recorded on a Waters QToF Premier Quadrupole instrument, in both positive and negative modes. Cyanomethyl esters of the α-hydroxy acids were prepared by reaction of the acid with chloroacetonitrile and triethylamine in dimethylformamide, and purified via silica gel chromatography using a 1:1 mixture of ethyl acetate and hexane^51^. Coupling of Iah/Fah/Lah/Vah to pCA was achieved by dissolving the cyanomethyl ester of the hydroxy acid (0.04 mmol) and pCA (5 mg, 0.008 mmol) in dry dimethylformamide (200 μl) in a screw-top vial (1 dram) and tetrabutylammonium acetate (30 mg, 0.1 mmol) was added. The solution was stoppered and stirred at room temperature and monitored by HPLC over several hours (2 µl aliquots were removed and diluted into 100 µl of a 4:1 mixture of A:B buffer prior to injection) with detection at 261 nm. Unreacted pCA elutes at ~9 min, and the α-hydroxy acid pCA product around 10–13 min. The reaction was judged complete after consumption of free pCA, after ~4 h. The product was isolated via preparative HPLC under the same gradient, and lyophilized overnight to a white residue. This material was carefully dissolved in ~50 µl dry dimethyl sulfoxide (DMSO) and checked for product integrity via HPLC and stock concentration was approximated on a NanoDrop UV–vis spectrometer using the known molecular extinction coefficient for pCA. Fah/Lah/Vah/Iah-pCA isolated in this manner had its concentration adjusted to 3 mM with DMSO and the stock was stored in aliquots at −28 °C. Mass spectrometry confirmed the identity of the molecule (Supplementary Figure 8).

Ligation of dinucleotide-amino acid substrates to tRNA: *Tetrahymena thermophila* (THG) and Pyrrolysine from *Barkeri fusaro* (Pyl) tRNAs lacking the last two nucleotides (-CA) were generated either by in vitro transcription^52^ or were chemically synthesized and HPLC purified by Integrated DNA Technologies, Inc. (Coralville, IA, USA). Folding was accomplished as follows: lyophilized pellets of tRNA were re-suspended in 10 mM HEPES, 3 mM MgCl_2_, pH 7.5, and then subjected to a thermocycler protocol with temperature at 94 °C for 3 min, followed by linear cooling to 4 °C over the course of the next 20 min. The tRNA ligation to the pCA dinucleotide was achieved with RNA T4 ligase and recovery of the full-length tRNA was performed by phenol–chloroform extraction and ethanol precipitation^52^.

### Two-electrode voltage clamp recordings

Ionic currents through the Shaker channels expressed in oocytes were recorded by TEVC carried out using an OC-725 voltage clamp amplifier (Warner). Recordings were carried out in high K^+^ solution (118 mM KCl, 0.5 mM CaCl_2_, 1 mM MgCl_2_, 5 mM HEPES-KOH, pH 7.4). Glass microelectrodes were backfilled with 3 M KCl and typically had resistances of 1–6 MΩ. Data were sampled at 10 kHz and filtered at 1 kHz. In most cases, leak currents were subtracted using a P/8 protocol^53^, except for pdCpA conditions wherein the small amount of leak current observed was not voltage dependent and therefore not subtracted. *G*–*V* curves were obtained by plotting the normalized tail current amplitudes 2–4 ms after switching the voltage, with the exception of the V369Vah Shaker mutant which shows rapid deactivation. For V369Vah and the V369Val native control, the *G*–*V* curves were determined by dividing the measured current amplitude at a given test voltage by the driving force. ∆∆*G* in Fig. 3b was calculated as (zFV_d_)aa −(zFV_d_)ah, where z and V_d_ are determined from Boltzmann fits and *F* is the Faraday constant. All data shown are mean ± standard deviation from 3 to 13 oocytes. Data were repeated from at least two separate batches of cRNA and suppressor tRNA.

Both the *Tetrahymena thermophila* (THG73)^54^ and Pyrrolysine (Pyl, from *Barkeri fusaro*)^55^ suppressor tRNAs were used in these studies. We tested THG73- and Pyl-tRNAs at all positions and for 5 of the 8 positions investigated, no difference was observed with either of these suppressor tRNAs. For F370TAG, we observed substantial bleed-through using the THG73-tRNA, while no bleed-through was observed when using the Pyl-tRNA. Therefore, for the F370TAG, only the data obtained using the using Pyl-tRNAs are reported. For the L358TAG, we did not observe any rescue using the Pyl-tRNA, but rescue was observed using the THG73-tRNA. We also observed much greater rescue at L375TAG position using THG73-tRNAs compared to the Pyl-tRNAs, and therefore for these two positions, only the data obtained using the THG73-tRNA are reported. For the other positions, the values reported combines the data obtained using the THG73- and the Pyl-tRNAs.

### Measurement of kinetics

In order to slow deactivation kinetics to more accurately compare rates between mutants, rate measurements for Supplementary Figure 3 were made in 100 mM RbCl, 0.3 mM CaCl_2_, 1 mM MgCl_2_, 10 mM HEPES-RbOH, pH 7.6^56^. Gating properties were similar in Rb^+^ conditions (Supplementary Table 2). Kinetic measurements of V369 variants were performed in Ringers solution (2 mM KCl, 116 mM NaCl, 0.5 mM CaCl_2_, 1 mM MgCl_2_, 5 mM HEPES-NaOH, pH 7.4) in order to resolve tail currents in the voltage range of 0 to −40 mV with an NPI Turbo TEC-03 amplifier with series resistance compensation engaged. In all cases, data were P/8 subtracted and tail currents were fit to a single exponential.

### Base hydrolysis of ester containing Shaker polypeptides

We introduced a Gly-Gly linker followed by the FLAG epitope (DYKDDDDK) at the C-terminal end of the *Shaker* WT, V363TAG, and V369TAG genes (Supplementary Table 3). Thirty to fifty oocytes were injected with either WT-FLAG *Shaker* cRNA or co-injected with the V363TAG-FLAG or V369TAG-FLAG cRNA and Pyl-tRNA-Vah and incubated at 12–16 °C. One to two days after injection, the oocytes were homogenized and a crude membrane preparation was carried out similarly to that previously described^57^. Briefly, the oocytes were homogenized on ice in 1 ml HEDP buffer (100 mM HEPES-NaOH, pH 7.5, 1 mM EDTA) with the added protease inhibitors: 0.5 µg/ml leupeptin, 1 µg/ml pepstatin A, 1 µg/ml aprotinin, and 0.5 mM phenylmethanosulfonyl fluoride. Homogenates were centrifuged at 3000 × *g* for 10 min at 4 °C. The supernatants were then overlaid on a 2.5-ml 15% sucrose cushion prepared in HEDP buffer and centrifuged at 175,000 × *g* for 1.5 h. The membrane pellet obtained was re-suspended in ~25 µl of 2× sodium dodecyl sufate (SDS) sample buffer (100 mM Tris-Cl, pH 6.8, 3.2% SDS, 0.04% Bromophenol Blue, 20% glycerol, 12.5 mM β-mercaptoethanol, 50 mM dithiothreitol).

Prior to base hydrolysis, the samples were heated at 70 °C for 30 min. to unfold the protein. Base hydrolysis was carried out using 0.7 M NaOH for 30 min at room temperature (RT). Base-treated samples were neutralized with 0.7 M HCl and heated at 70 °C for 5 min before loading on a 10% SDS-polyacrylamide gel electrophoresis. Following electrophoresis, the proteins in the gel were transferred onto a 0.45 µm polyvinylidene difluoride membrane using 25 mM Tris, 192 mM glycine, 20% methanol, and 0.1% SDS as the transfer buffer. The membranes were probed with an anti-FLAG M2 primary antibody, dilution 1:1000 (Sigma catalog # F1804) and a goat anti-mouse IgG (H + L)-horseradish peroxidase conjugate secondary antibody, dilution 1:8333 (Bio-Rad catalog # 1706516) and developed using the SuperSignal West Femto Maximum Sensitivity Substrate Kit (ThermoFisher Scientific, catalog # 34094). Exposure time was 0.5 s. Western blot results were repeated from at least 2–3 separate batches of oocytes. A full uncropped blot of the channel hydrolysis experimental data shown in Fig. 2, including markers, is provided in Supplementary Figure 9.

### Gating charge measurements

Capacitive currents were recorded in oocytes from *Shaker* proteins bearing the W434F mutation which essentially eliminates ionic current through the pore of the channel without altering voltage-sensor movement^37^. Injection conditions were as follows: for W434F *Shaker*, 25 ng of cRNA was injected and recordings were made 24 h later. For encoding of valine or α-hydroxy-valine, 25 ng of W434F/V363TAG or W434F/V369TAG cRNA was co-injected with ~150 ng of Pyl-valine or Pyl-α-hydroxy-valine tRNA and recordings were made 24 to 36 h later. In parallel experiments, we co-injected each cRNA with ~150 ng of Pyl-pdCpA (the full length, but unacylated tRNA) to check for gating currents arising from spurious read-through of the introduced TAG codons. All recordings were made in a solution which contained 100 mM *N*-methyl-d-glucamine-Cl, 0.3 mM CaCl_2_, 1.0 mM MgCl_2_, and 5 mM HEPES (pH 7.6). Cells were held at −100 mV and typically pulsed through the range of −150 to +100 mV. The length of the test pulse was 30 ms. For analysis, the leak-subtracted OFF capacitive currents were integrated over time, and the linear capacitance (calculated using linear regression of the integrated signals from +50 to +70 mV) was subtracted to isolate nonlinear gating *Q* (Supplementary Figure 5)^38,39^. The nonlinear charge displacement from the negative controls (which represents the sum of all potential sources of contamination of *Q*) was negligible (Supplementary Figure 6). *Q*–*V* curves generated from experiments utilizing 5 mV depolarization steps were fit to a double Boltzmann function in Origin Pro (OriginLab, Northhampton, MA, USA).

### MD simulation

The crystal structure of a K_V_1.2/2.1 chimera channel (pdb:2R9R) was used as the model for MD simulations. The transmembrane protein (without the cytoplasmic T1 domain) together with crystal water molecules and the four potassium ions bound to the selectivity filter were embedded in a pure POPC lipid bilayer and solvated with 0.15 M of KCl and TIP3P water^58^ using CHARMM-GUI Membrane Builder^59^. In addition to the WT channel, an α-hydroxy was introduced to the S4 backbone at I291 or I297 (V363 or V369 in a *Shaker* channel) sites, producing three independent systems with dimensions 120 Å × 120 Å × 105 Å.

### Simulation protocol

All three systems first underwent 10,000 steps of minimization (using conjugate gradient algorithm) followed by 5 ns isothermal–isobaric ensemble (NPT) equilibration with positional harmonic restraints on all heavy atoms of the protein (*k* = 10 kcal/mol/Å^2^ for the first 1 ns, *k* = 1 kcal/mol/Å^2^ for the remaining 4 ns). Thereafter, 15 ns of restraint-free NPT equilibration were performed. The last frame of the NPT equilibration was then used for four independent (different initial velocities) in-plane area ensemble (NPAT) simulations (constant membrane area) for each system. Each independent trajectory was simulated for 150 ns and the last 100 ns were used for the analysis. All simulations were carried out with NAMD 2.12^60^, using CHARMM36m protein^61^ and CHARMM36 lipid^62^ parameters. Periodic boundary condition and a nonbonded cutoff of 12 Å (with 10 Å switching distance and vdW force switching) were used. Long-range electrostatics were calculated using the particle mesh Ewald method^63^ with 1 Å grid spacing. Two femtosecond time steps were employed with SHAKE algorithm^64^ to constrain hydrogen bond lengths. Constant temperature of 310 K was maintained by Langevin thermostat^65^ with a damping coefficient of 1 ps^−1^. Nosé-Hoover Langevin piston^66^ with a period of 100 ps and decay time of 50 ps was employed to maintain constant pressure at 1 atm.

### Parameterization of backbone α-hydroxy

Parameterization of the protein backbone α-hydroxy was mainly done by analogy to existing CHARMM36 force field parameters. Partial charges of the backbone α-hydroxy were taken directly from the ester bond in lipids. The initial bond, angle, and dihedral parameters were generated by analogy using the CGenFF webserver^67^. The results were satisfying, with only one angle and three dihedral terms receiving penalty scores between 10 and 40. These terms were further refined using the FFTK plugin in VMD^68^. Partial charges and other parameters of the side-chain atoms were not modified.

## Electronic supplementary material

Supplementary Movie 3

Supplementary Movie 2

Supplementary Movie 1

Supplementary Information

Description of Additional Supplementary Files

Reporting Summary

## Data Availability

Data supporting the findings of this manuscript are available from the corresponding authors upon reasonable request. A Reporting Summary for this Article is available as a Supplementary Information file.
