# Supplementary material for: Main-chain mutagenesis reveals intrahelical coupling in an ion channel voltage-sensor
Source: Nat Commun. 2018 Nov 29;9:5055. doi: 10.1038/s41467-018-07477-3 (PMC6265297; doi:10.1038/s41467-018-07477-3)
Supplement: Supplementary file 4 — Supplementary Information [file 41467_2018_7477_MOESM4_ESM.pdf]

Main-chain mutagenesis reveals intrahelical coupling in an ion channel voltage-sensor

Infield, Matulef et al

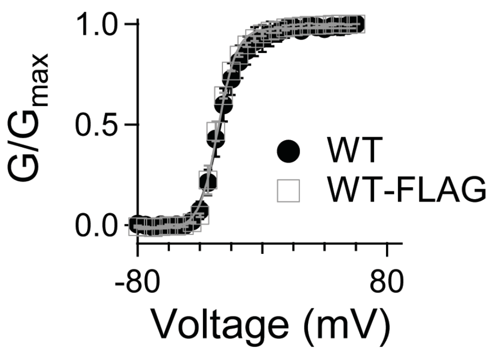

Supplementary Figure 1 Conductance-voltage relationships for WT *Shaker* and C-terminally FLAG tagged channels.

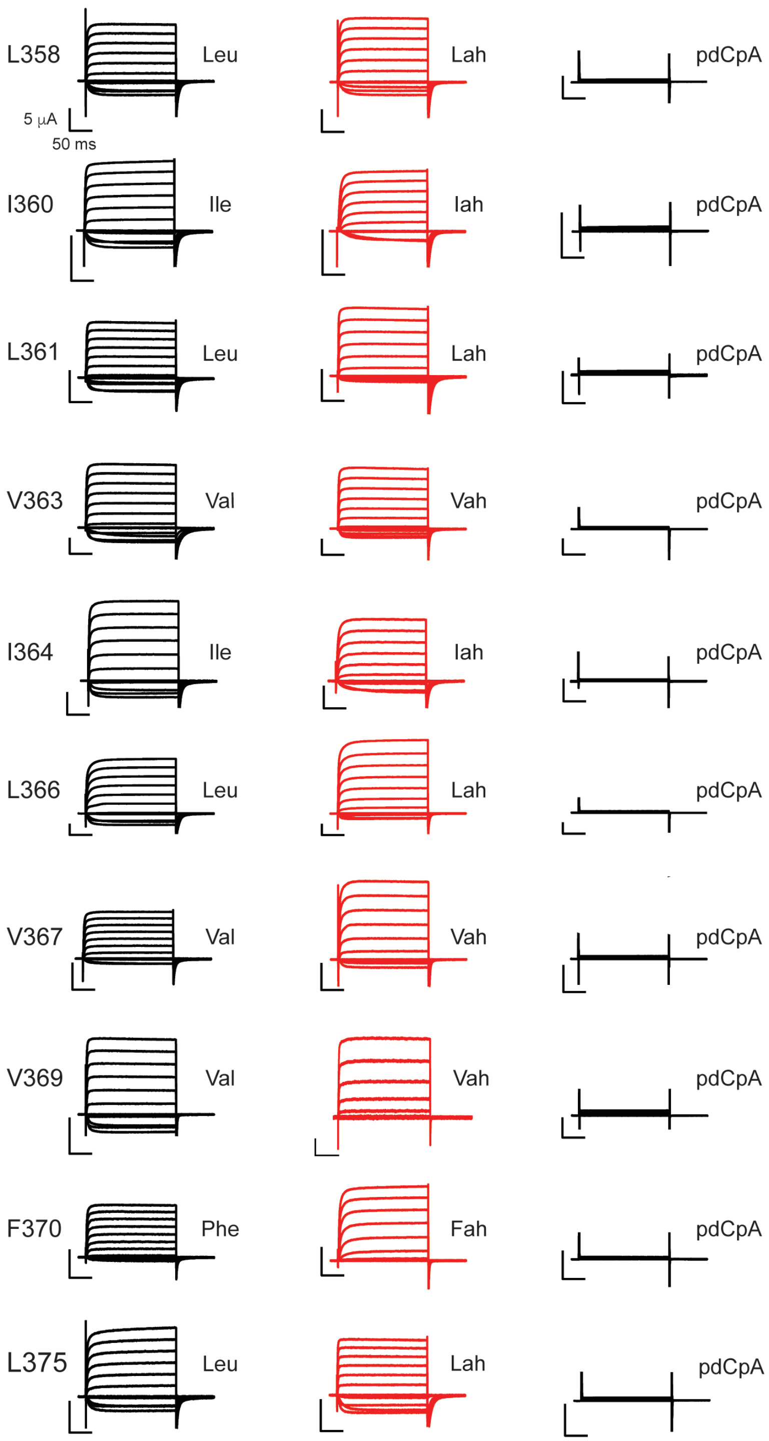

Supplementary Figure 2 Representative current traces as described in Figure 3 for rescue with amino acids or alpha-hydroxy acids. The negative control (pdCpA) condition consists of the oocyte injected in parallel with *Shaker* TAG cRNA and a full-length unacylated tRNA, see Methods and text for additional experimental detail.

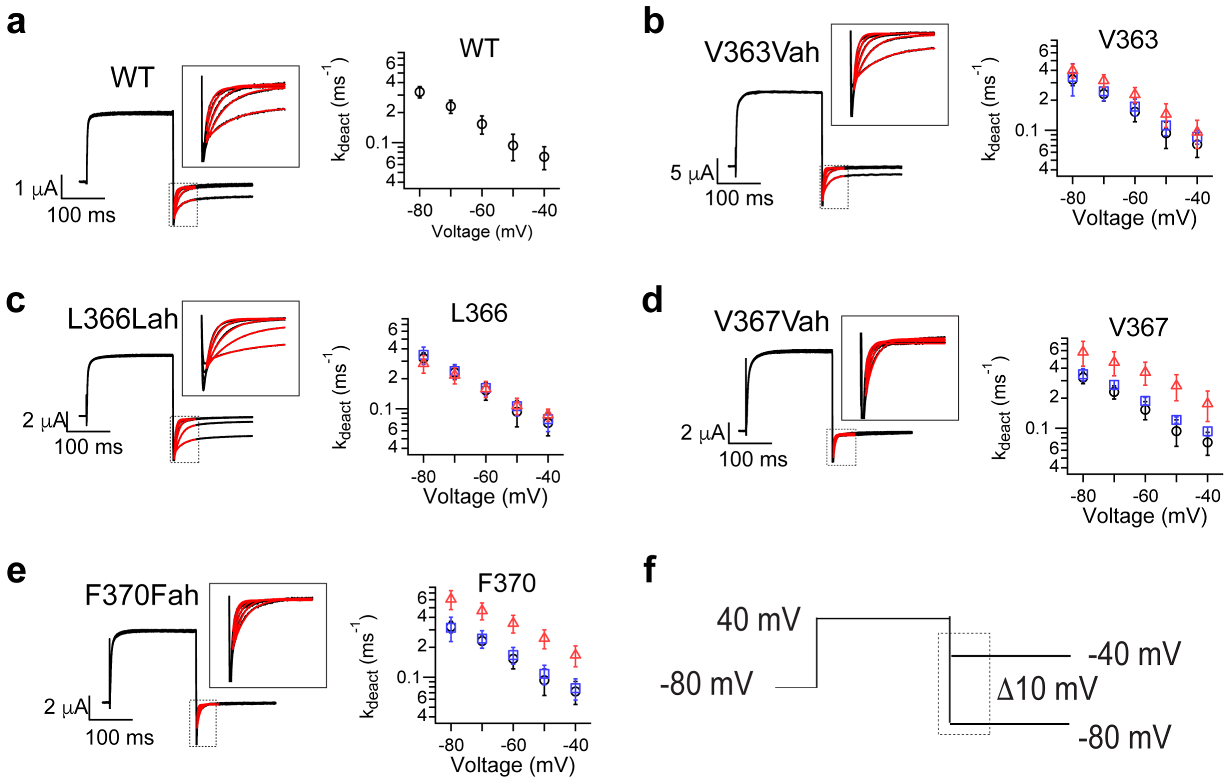

Supplementary Figure 3 Deactivation kinetics of the indicated channel types. **a-e** Representative current traces measured in response to the voltage protocol shown in (**f**). Recordings were made in 100 mM $RbCl_2$, 1 mM $MgCl_2$, 0.3 mM $CaCl_2$, and 10 mM HEPES-RbOH pH 7.6. Fits to single exponentials are shown in red, with the insets showing the boxed region of the traces. In each example, representative data are shown with the inset highlighting the fit (red), with the voltage-dependent rates of deactivation (mean ± standard deviation from 5-12 oocytes). WT- black circles, amino acids (Val,Phe,Leu)- blue squares, and alpha-hydroxy acids (Vah, Fah, and Lah)- red triangles.

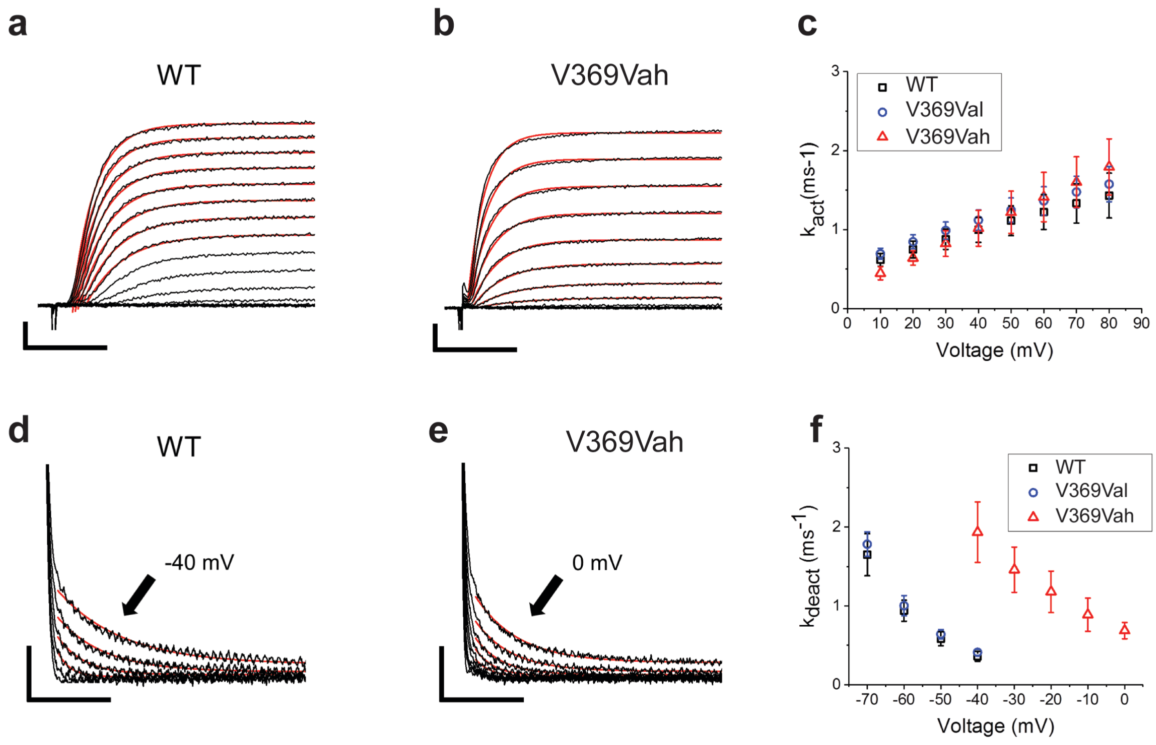

Supplementary Figure 4 Kinetic analysis of the Shaker V369Vah mutant. **a**,**b**, Example traces showing kinetics of opening of WT or V369Vah Shaker. Oocytes were held at -80 mV and pulsed from -80 to +80 mV in 10 mV increments for a duration of 100 ms. Activation time course was fit to a single exponential for the voltage range of +10 mV to +80 mV. Scale bars: vertical- 5 μA, horizontal 3 ms. **c** Summary data for activation, n=6-11. **d,e** Example traces showing kinetics of deactivation of WT and of V369Vah Shaker. Oocytes were held at -80 mV, pulsed to +50mV for 100 ms, then pulsed from -40 to -80 mV (WT, Val) or from -0 to -80 mV (Vah) in 10 mV increments, as shown in the panels. Deactivation time course was fit to a single exponential (**f**). All scale bars: vertical- 5 μA, horizontal 3 ms. Summary data for deactivation, n=6-11.

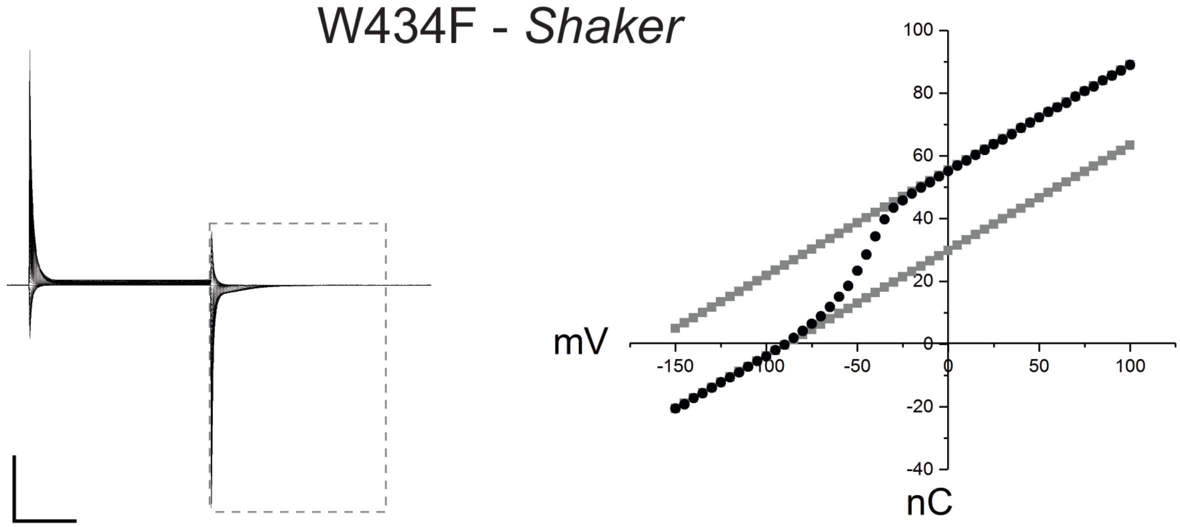

Supplementary Figure 5 Method for determining macroscopic gating currents. **a** Example trace for W434F-*Shaker* recordings from oocytes which contain linear (membrane capacitance) and non-linear (voltage-sensor gating) current components. Oocytes were held at -80 mV and pulsed from -150 mV to 100 mV in 5 mV increments. Scale bar: 40 μA vertical, 10 ms horizontal. The OFF capacitive currents within the time range indicated by the dashed box integrated over time are inverted in sign and plotted to the right as a function of test voltage (black data points). **b** The linear component of the capacitance (gray data points) was subtracted from the total signal to yield the nonlinear Q for each voltage.

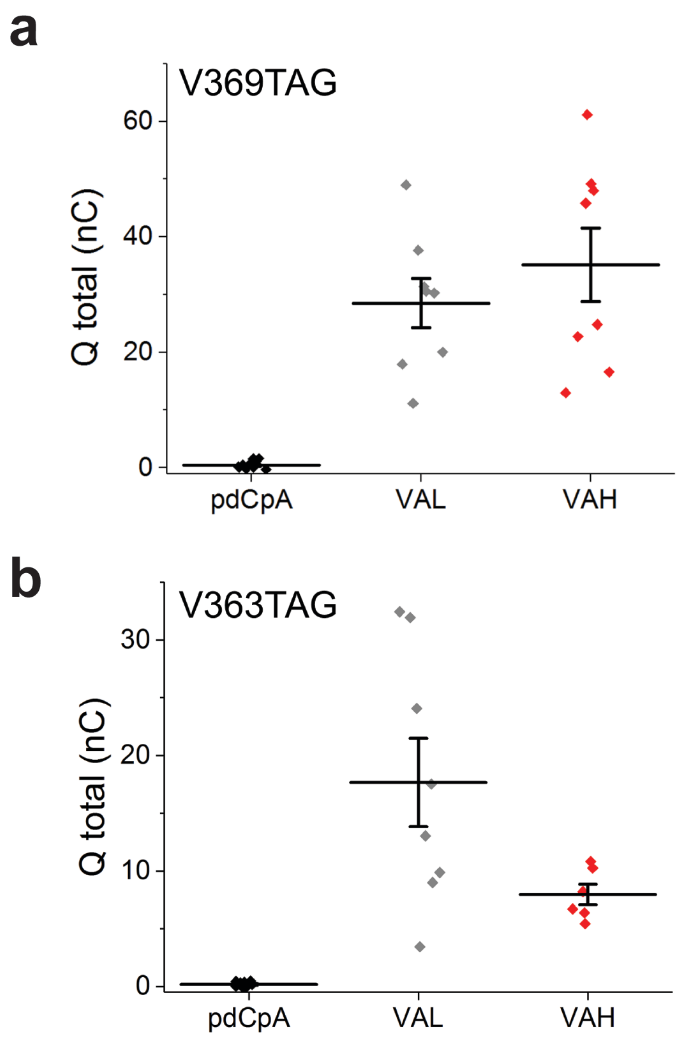

Supplementary Figure 6 Total gating current (Q). In all cases, Q (total) was derived at 0 mV from integration of the OFF currents after subtraction of linear capacitance (Supplementary Figure 5). The quantified Q in the pdCpA- ligated (full length tRNA) controls therefore represent the combined potential contamination from bleedthrough and nonlinearity in oocyte capacitance. **a** Integrated gating current signal generated by co-injection of Shaker W434F-V369TAG cRNA with either unacylated (pdCpA) or acylated (valine or valine-alpha hydroxy) tRNA. **b** Same as in (**a**), but for the V363 position. Brackets +/- Std dev.

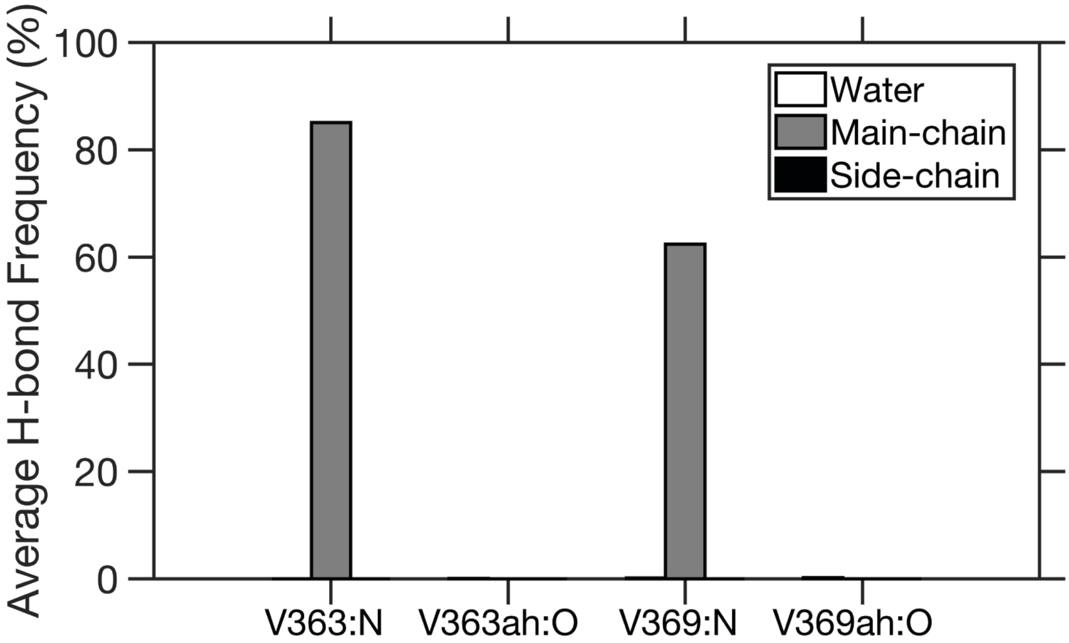

Supplementary Figure 7 Hydrogen bond frequency at V363 and V369 in MD simulations. H-bond forming frequency was calculated between the main-chain amide nitrogen atom/ester oxygen atom and all other heavy atoms, using distance and angle cutoff of 3.5 Å and 4.5 Å , respectively. Contacts with water or side-chains from the V363 and V369 main-chain amide accounted for less than 1% of interactions. The V369 amide H-bond samples multiple conformations, two of which (turn, coil) lack H-bonding, thus average H-bond frequency is less that 100%. All frequencies are shown in percentage, i.e. average over all 4 VSDs and the whole MD trajectory.

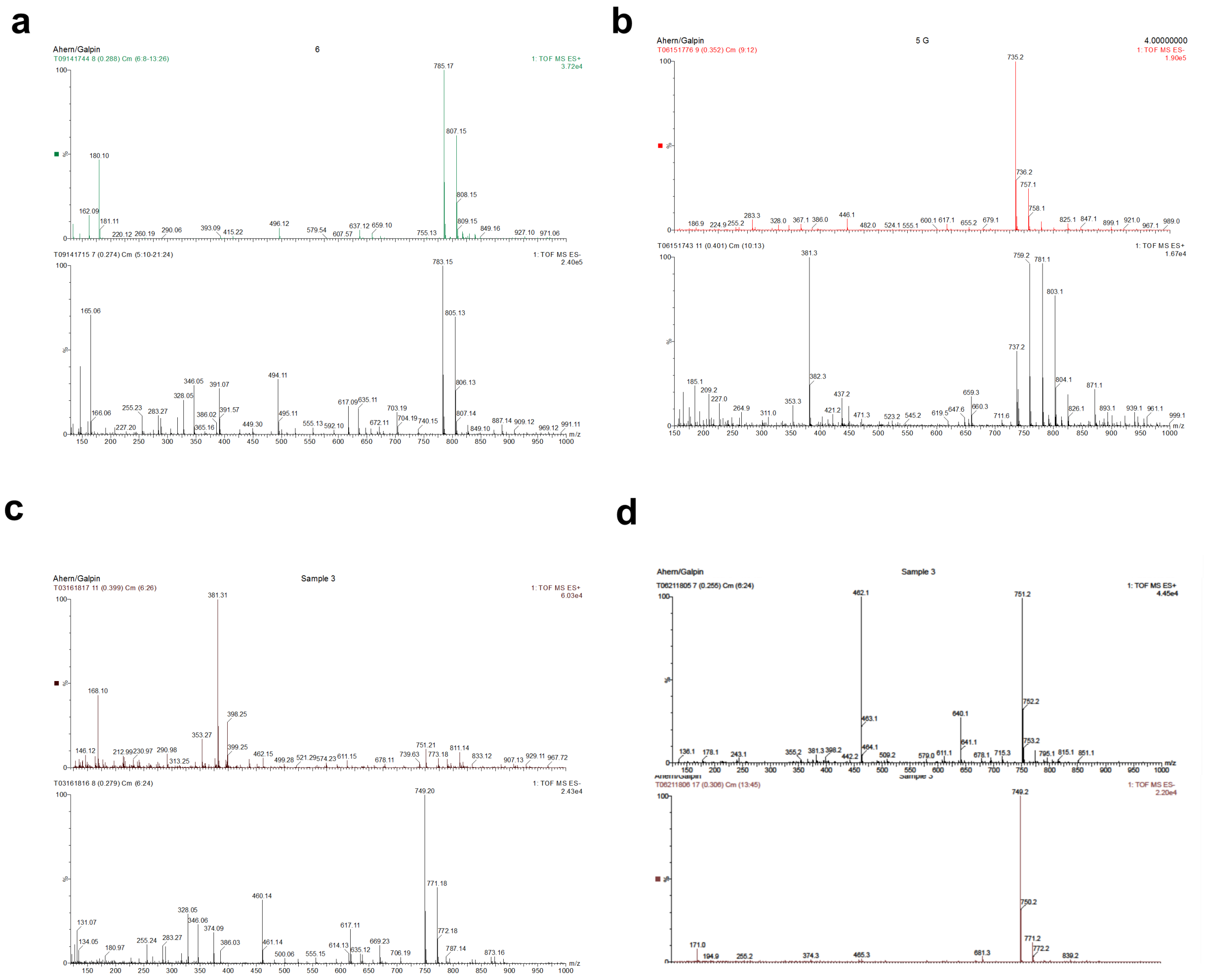

Supplementary Figure 8 Mass spectra confirming synthesis of the alpha hydroxyl acid-dincuelotide substrates used in this study. **a**, LRMS (+/- mode) of FAH-pCA. Calc. mass for $C_{28}H_{34}N_8O_{15}P_2$ 784.16. **b**, LRMS (+/- mode) of VAH-pCA. Calc. mass for $C_{24}H_{34}N_8O_{15}P_2$ 736.16. **c**, LRMS (+/- mode) of LAH-pCA. Calc. mass for $C_{25}H_{36}N_8O_{15}P_2$ 750.18. **d**, LRMS (+/- mode) of IAH-pCA. Calc. mass for $C_{25}H_{36}N_8O_{15}P_2$ 750.18.

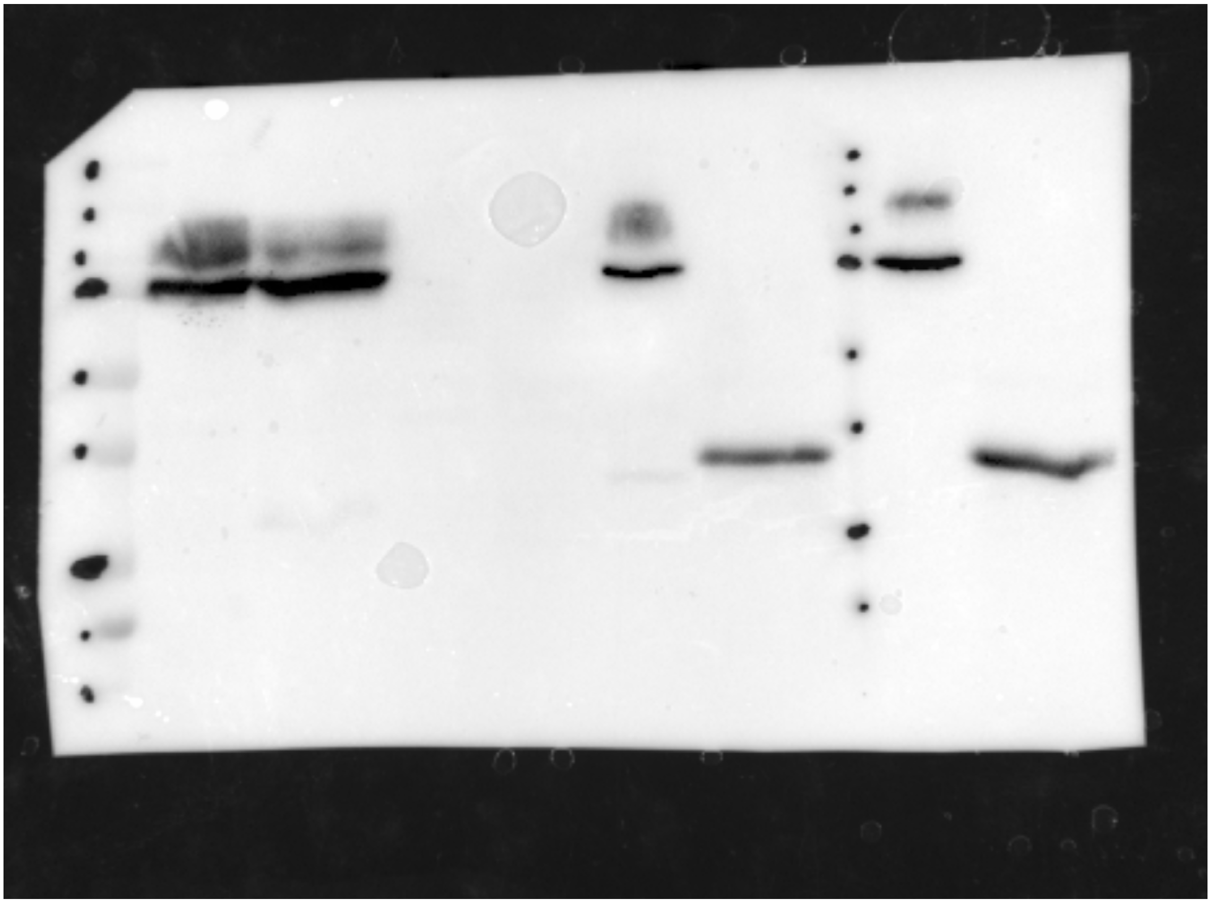

Supplementary Figure 9 Uncropped full blot from Figure 2, including markers on left. Sample labeling as shown in Figure 2.

Supplementary Table 1: QV Parameters

Variant	Frac.Q1 /Q2	Q1					Q2			
		Stdev	V(1/2)	Stdev	Z	Stdev	V(1/2)	Stdev	Z	Stdev
W434F-*Shaker* (5)	0.32/68	0.06	-68.1	7.9	2.0	0.33	-45.4	2.3	4.3	0.66
W434F-V363Val (4)	0.29/71	0.11	-73.4	2.2	2.2	0.52	-44.5	2.4	3.8	0.10
W434F-V363Vah (4)	0.22/78	0.05	**-103.4**	4.6	1.6	0.25	**-52.3**	2.1	**2.5**	0.15
W434F-V369Val (8)	0.27/73	0.08	-66.9	11.7	1.8	0.24	-44.7	4.5	4.1	0.37
W434F-V369Vah (8)	**0.79/21**	0.03	-74.5	5.3	**0.87**	0.0	**-2.4**	3.0	**2.3**	0.33

Bold = $p<0.01$, relative to the respective native rescue for each position.

Supplementary Table 2 Gating parameters with Extracellular Rb^+

	V_d	z		V_d	z
WT	-35.9 ± 1.9 (10)	4.5 ± 0.8 (10)			
V363Val	-33.8 ± 2.7 (8)	4.0 ± 0.7 (8)	V363Vah	-29.6 ± 4.1 (8)	2.7 ± 0.5 (8)
L366Leu	-32.9 ± 1.4 (5)	3.7 ± 0.5 (5)	L366Lah	-32.1 ± 3.9 (11)	2.6 ± 0.4 (11)
V367Val	-34.6 ± 2.6 (5)	4.7 ± 0.9 (5)	V367Vah	-8.1 ± 3.2 (8)	2.7 ± 0.3 (8)
F370Phe	-33.1 ± 2.5 (11)	4.0 ± 0.8 (11)	F370Fah	5.3 ± 2.5 (10)	3.1 ± 0.2 (10)

Supplementary Table 3 Oligo Sequences

L358TAG-For	5'-GAATCAGGCTATGTCCTAGGCAATATTACGAGTG-3'
L358TAG-Rev	5'-CACTCGTAATATTGCCTAGGACATAGCCTGATTC-3'
I360TAG-For	Generated by Gibson cloning, see below and Methods
L361TAG-For	5'-CTATGTCCTTGGCAATATAGCGAGTGATACGATTAG-3'
L361TAG-Rev	5'-CTAATCGTATCACTCGCTATATTGCCAAGGACATAG-3'
V363TAG-For	5'-GCTATGTCCTTGGCAATATTACGATAGATACGATTAGTTCGAGTATTTCGAATATTTAAG-3'
V363TAG-Rev	5'-CTTAAATATTCGAAATACTCGAACTAATCGTATCTATCGTAATATTGCCAAGGACATAGC-3'
I364TAG-For	Generated by Gibson cloning, see below and Methods
L366TAG-For	5'-CAATATTACGAGTGATACGATAGGTTCGAGTATTTCGAATATTTAAG-3'
L366TAG-Rev	5'-CTTAAATATTCGAAATACTCGAACCTATCGTATCACTCGTAATATTG-3'
V367TAG-For	5'-GGCAATATTACGAGTGATACGATTATAGCGAGTATTTCGAATATTTAAGTTATCTAGG-3'
V367TAG-Rev	5'-CCTAGATAACTTAAATATTCGAAATACTCGCTATAATCGTATCACTCGTAATATTGCC-3'
V369TAG-For	5'-GGCAATATTACGAGTGATACGATTAGTTCGATAGTTTCGAATATTTAAGTTATCTAGGC-3'
V369TAG-Rev	5'-GCCTAGATAACTTAAATATTCGAAACTATCGAACTAATCGTATCACTCGTAATATTGCC-3'
F370TAG-For	5'-GTGATACGATTAGTTCGAGTATAGCGAATATTTAAGTTATCTAG-3'
F370TAG-Rev	5'-CTAGATAACTTAAATATTCGCTATACTCGAACTAATCGTATCAC-3'
L375TAG-For	5'-GTATTTCGAATATTTAAGTAGTCTAGGCATTCGAAGGG-3'
L375TAG-Rev	5'-CCCTTCGAATGCCTAGACTACTTAAATATTCGAAATAC-3'
FLAG-For	5'-GGTGGTGATTATAAGGATGATGATGATAAGTGACTACTGGTGCAAAAG-3'
FLAG-Rev	5'-CTTATCATCATCATCCTTATAATCACCACCAACGTCGGTCTCGATACTAAC-3'

The following mutants were assembled by Gibson cloning into the naturally occurring xbaI-bsteII sites, see Methods.

I360TAG
Gttatattgctatcaattgttatattttgtctagaaacattacccgaatttaagcattacaagg
tgttcaatacaacaacaaatggcacaaaaatcgaggaagacgaggtgcctgacatcacagatcc
tttcttccttatagaaacgttatgcattatttggtttacatttgaactaactgtcaggttcctc
gcatgtccgaacaaattaaatttctgcagggatgtcatgaatgttatcgacataatcgccatca
ttccgtactttataacactagcgactgtcgttgccgaagaggaggatacgttaaatcttccaaa
agcgccagtcagtccacaggacaagtcatcgaatcaggctatgtccttggca**TAG**ttacgagtg
atacgattagttcgagtatttcgaatatttaagttatctaggcattcgaagggtttacagatct
taggacgaactctgaaagcctcaatgcgggaattaggtttacttatatttttcttatttatagg
cgtcgtactcttctcatcggcggtttattttgcggaagctggaagcgaaaattccttcttcaag
tccatacccgatgcattttggtgggcggtggttaccatgaccaccgttggatatggtgacatg

I364TAG
gttatattgctatcaattgttatattttgtctagaaacattacccgaatttaagcattacaagg
tgttcaatacaacaacaaatggcacaaaaatcgaggaagacgaggtgcctgacatcacagatcc
tttcttccttatagaaacgttatgcattatttggtttacatttgaactaactgtcaggttcctc
gcatgtccgaacaaattaaatttctgcagggatgtcatgaatgttatcgacataatcgccatca
ttccgtactttataacactagcgactgtcgttgccgaagaggaggatacgttaaatcttccaaa
agcgccagtcagtccacaggacaagtcatcgaatcaggctatgtccttggcaatattacgagtg
TAGcgattagttcgagtatttcgaatatttaagttatctaggcattcgaagggtttacagatct
taggacgaactctgaaagcctcaatgcgggaattaggtttacttatatttttcttatttatagg
cgtcgtactcttctcatcggcggtttattttgcggaagctggaagcgaaaattccttcttcaag
tccatacccgatgcattttggtgggcggtggttaccatgaccaccgttggatatggtgacatg
