# Supplementary material for: Main-chain mutagenesis reveals intrahelical coupling in an ion channel voltage-sensor
Source: Nat Commun. 2018 Nov 29;9:5055. doi: 10.1038/s41467-018-07477-3 (PMC6265297; doi:10.1038/s41467-018-07477-3)
Supplement: Supplementary file 5 — Description of Additional Supplementary Files [file 41467_2018_7477_MOESM5_ESM.docx]

**Title:** Supplementary Movie 1 (Kv_WT.mp4)
**Description:** Secondary structure dynamics of S4 helix in WT Kv1.2/2.1 channel. S4 main-chain structure is represented by the transparent cartoon overlay and main-chain H-bonds are indicated by green lines. Color codes for secondary structure: helix - gray, 3-10 helix - blue, turn - orange, coil - white.

**Title:** Supplementary Movie 2 (Kv_V363Vah.mp4)
**Description:** Secondary structure dynamics of S4 helix in V363Vah Kv1.2/2.1 channel. Same color codes as Supplementary Movie 1. Ester oxygen atom of V363 is represented by the red sphere.

**Title:** Supplementary Movie 3 (Kv_V369Vah.mp4)
**Description:** Secondary structure dynamics of S4 helix in V363Vah Kv1.2/2.1 channel. Same color codes as Supplementary Movie 1. Ester oxygen atom of V369 is represented by the red sphere.
